# Clustering of countries according to the COVID-19 incidence and mortality rates

**DOI:** 10.1186/s12889-022-13086-z

**Published:** 2022-04-01

**Authors:** Kimiya Gohari, Anoshirvan Kazemnejad, Ali Sheidaei, Sarah Hajari

**Affiliations:** 1 Department of Biostatistics, Faculty of Medical Sciences, Tarbiat Modares University, P.O. BOX 14115-111, Tehran, Iran; 2 Department of Epidemiology and Biostatistics, School of Public Health, Tehran University of Medical Sciences, Tehran, Iran; 3 Department of Computer Science, University of the Sunshine Coast, Sippy Downs, Queensland Australia

**Keywords:** COVID-19, Trajectory, Clustering, Mortality rate, Incidence rate

## Abstract

**Background:**

Two years after the beginning of the COVID-19 pandemic on December 29, 2021, there have been 281,808,270 confirmed cases of COVID-19, including 5,411,759 deaths. This information belongs to almost 216 Countries, areas, or territories facing COVID-19. The disease trend was not homogeneous across these locations, and studying this variation is a crucial source of information for policymakers and researchers. Therefore, we address different patterns in mortality and incidence of COVID-19 across countries using a clustering approach.

**Methods:**

The daily records of new cases and deaths of 216 countries were available on the WHO online COVID-19 dashboard. We used a three-step approach for identifying longitudinal patterns of change in quantitative COVID-19 incidence and mortality rates. At the first, we calculated 27 summary measurements for each trajectory. Then we used factor analysis as a dimension reduction method to capture the correlation between measurements. Finally, we applied a K-means algorithm on the factor scores and clustered the trajectories.

**Results:**

We determined three different patterns for the trajectories of COVID-19 incidence and the three different ones for mortality rates. According to incidence rates, among 206 countries the 133 (64.56) countries belong to the second cluster, and 15 (7.28%) and 58 (28.16%) belong to the first and 3rd clusters, respectively. All clusters seem to show an increased rate in the study period, but there are several different patterns. The first one exhibited a mild increasing trend; however, the 3rd and the second clusters followed the severe and moderate increasing trend. According to mortality clusters, the frequency of sets is 37 (18.22%) for the first cluster with moderate increases, 157 (77.34%) for the second one with a mild rise, and 9 (4.34%) for the 3rd one with severe increase.

**Conclusions:**

We determined that besides all variations within the countries, the pattern of a contagious disease follows three different trajectories. This variation looks to be a function of the government’s health policies more than geographical distribution. Comparing this trajectory to others declares that death is highly related to the nature of epidemy.

## Introduction

In December 2019, the municipal health commission of Wuhan city, Hubei province, China, issued an urgent notice Because of an outbreak of viral pneumonia due to unknown causes. The gene sequencing conducted by the China Health Commission expert group revealed that the disease had been caused by a novel severe acute respiratory syndrome coronavirus 2 (SARS-CoV-2) [1]. The World Health Organization (WHO) officially called it the novel coronavirus “2019-ncov” (2019 novel coronavirus), On January 12 [2].

On January 30, 2020, the WHO reported the first confirmed 2019-nCoV acute respiratory disease cases in Finland, India, and the Philippines; all had travel history to Wuhan city. In this way, the disease rapidly spreads worldwide and has been the foremost priority and concern of health systems [2]. In this regard, the WHO declared the COVID-19 as a global pandemic on March 10, 2020 [2].

Globally, as of 4:14 pm CET, December 29, 2021, there have been 281,808,270 confirmed cases of COVID-19, including 5,411,759 deaths, reported to WHO. This information belongs to almost 216 Countries, areas, or territories facing COVID-19 in these 3 years [3]. On the other hand, there is significant heterogeneity between countries and regions in related statistics. For instance, there were 102,287,397 confirmed cases in the Americas (As the WHO region) compared with only 7,164,485 confirmed cases in Africa at the end of 2021. Another example could be the comparison between adjacent European countries. In the same time interval, the incidence of COVID-19 in Sweden was estimated to be 12,730.79 new cases per 100,000 population, whiles its neighboring country Norway confirmed 7439.44 new cases. Controlling the disease and reducing its burden is possible if we justify the sources of these variations [3].

Many studies aim to address the sources of variations in epidemiological patterns and behaviors of COVID-19 across societies. They explored the different aspects from age-sex distribution [4, 5], the prevalence of risk factors and comorbidities [6], environmental factors [7], governments policies [8, 9], health system infrastructures [7], social adherence to protocols [10], Etc. Reviewing these studies reveals that the COVID-19 pandemic and its causes and consequences are not a simple phenomenon that we could summarize in single research [6, 11]. Therefore, we need to simplify the issues in a stepwise approach. The cornerstone of the following steps could be counting and diagnosing different patterns in COVID-19 incidence and mortalities.

Many data scientists have shown their tendency to study in this field because of publicly available data and its importance. One appealing and practical data-driven solution is clustering the countries according to their similarity in COVID-19 incidence and fatality rate [11]. Most studies in this context focused on temporal trends [12–15], and some others detected spatial patterns [1, 16–20] or hotspots [21–24]. Among all the clustering algorithms, the K-means algorithm has shown better performance than the others, including hierarchical, Fuzzy, K-medoids, C-means, and even model-based methods [11, 25–27].

The K-means algorithm clusters subjects according to the similarity of their features using a distance metric, usually the Euclidean distance. Therefore, feature selection is an essential step in this method. Some studies ignored the temporal trend and quickly applied the cumulative number of cases or deaths due to COVID-19 at a fixed time [8, 28, 29]. The other studies added the socioeconomic variables and health system metrics into the algorithm to enhance the clustering functionality [7]. Nevertheless, none of them correctly address the temporality issue. On the other hand, the trajectories of the cumulative number of cases are highly similar and S-shape. Therefore, differentiation between the countries in this subject is not easy to handle.

In this study, we propose an approach that includes three steps for clustering country-specific COVID-19 incidence and mortality trends. In summary, this method extracts the essential features of a trajectory and considers them in a K-means clustering algorithm. We divide the trend lines into measurements that describe different aspects of a trend at the first step. Then a dimension reduction method is applied to the measurements, and finally, a K-means algorithm clusters them [30, 31]. We conduct this approach for incidence and mortality rates separately and finally compare them.

### Data source

We extracted the daily new cases and mortality of COVID-19 for 220 countries and territories from the online available WHO COVID-19 dashboard. This information was gathered from official health organizations around the world [3]. The study period started from January 3, 2020, to August 28, 2021.

We saw a relatively high level of data fluctuations in daily trajectories in the data preparation. Besides, some countries corrected their reports after several days. Therefore, we prepared to aggregate daily incidence and death to the weekly summation.

As the start of epidemy differs across countries, we shifted the time origin into the date of reporting the first COVID-19 death. We considered a year equal to 54 weeks, so a trajectory started from the first COVID-19 death and continued to the year (54 weeks) after this origin date for each country. In this manner, we eliminate nuisance and non-informative variations in the initiation of the pandemic in the very early days.

A few countries reported data in low-quality levels, including large vales of incompleteness or huge daily variation. Some others had zero-inflated data in lots of days. These Issues were nonrelevant to the pattern of COVID-19 itself, so we ignore them in further analysis. Additionally, several countries had less than 54 weeks of data, so we ignored them too. Finally, we used 206 one-year length trajectories for the 206 different countries and territories in this study.

### Statistical methods

We used a three-step approach proposed by Leffondre et al. for identifying longitudinal patterns of change in quantitative health indicators [31]. We calculated 27 measurements in 4 classes for each trajectory at the first step. These measurements comprehensively summarize different aspects of a trajectory. The measurements related to a trajectory could be correlated. The meaning of correlation is the redundancy of measuring or emphasizing some aspects of a trajectory more than others. In this manner, we used factor analysis as a dimension reduction method to capture the correlation in the second step. Finally, we applied a K-means algorithm on the factor scores and clustered the trajectories.

The first class of measurements includes the ten elementary ones. In this class, we considered the range, mean over time, standard deviation, and coefficient of variation. These measures ignore the temporal trend of data but give essential information about the whole trajectory attributes. Other measurements in this class are based on the most detailed description of variation across time. The change is equal to the difference of last and first observations quantify the whole variation after all the duration of the period. In addition, the mean of change per week, the proportion of change to the first observation, and the proportion of change to mean of observation over time are derived from change measurements. Finally, a simple linear regression of observation on time directly evaluates the linear trend of observation. The two most essential components in this model are the slope of the line and the R square of the model. We considered both of these measures in the first class.

In contrast to the first class that only considered a linear relationship, class 2 focuses on nonlinearity and inconsistency of changes. This class introduces the first-order delta representing the change between two consecutive observations in a trend. This delta is the basic measurements of this class. The maximum, standard deviation, standard deviation per week, mean of absolute values, maximum of absolute value, the ratio of maximum to mean over time, the ratio of maximum to the slope of change, and the ratio of standard deviation to the slope of changes are other functions of the first-order delta that are introduced in this class.

Class 3 includes the measurements sensitive to nonmonotonicity and abrupt short-term fluctuation. This sensitivity is based on measuring the difference between two consecutive first-order differences. This class also includes the other function of this measure: mean, the mean of absolute values, maximum of absolute values, the ratio of maximum to mean over time, and ratios of maximum and mean to the mean of the absolute first element of difference.

The final class consists of 3 measures constructing early versus later change. We chose the year’s median as the generic cut-off point as a conservative approach. Using this definition, we also calculated the ratios of early to later change, early to total change, and late to the total change. The list of all measurements in classes is available in Table 1.

**Table 1:** Measures of summarizing the different aspects of a trajectory

Class	Measure	Note
1. Elementary measures of change	1. Range	Basic descriptive statistics that ignore the temporal trend
	2. Mean-over-time	
	3. Standard deviation (SD)	
	4. Coefficient of variation	
	5. Change	Last observation - First observation
	6. Mean change per week	Weekly value of change
	7. Change/first score	Rescale change based on the first observation
	8. Change/mean-over-time	Rescale the change based on all of the observations
	9. slop b	Achieve from a linear regression model of observations on times
	10. R2 of the linear model	The proportion of variation of observation that could be explained by the linear trend of the time
2. Measures of nonlinearity and in-constituency of change	11. Max Δ1 (Δ_1, i_ = y_i + 1_ - y_i_)	indicates that there is at least one big increase between two consecutive scores
	12. SD Δ_1_	A high value of measure indicates that the first differences are not constant and that the pattern is therefore not linear
	13. SD Δ_1_ per week	Adjust for the time elapsed between two consecutive scores
	14. Mean | Δ_1_|	A high value of measure indicates that there are many abrupt changes during the time
	15. Max | Δ_1_|	A high value of measure indicates that there is at least one crucial abrupt change regardless of the sign
	16. Ratio, max | Δ_1_|/mean-over-time	To measure the relative importance of the significant abrupt
	17. ratio, max | Δ_1_|/b	b is the coefficient of linear regression
	18. Ratio, SD Δ_1_/b	To assess the relative importance of the variability of the first differences
3. Measures sensitive to nonmonotonicity and abrupt short-term fluctuations	19. Mean Δ_2_ (Δ_2, i_ = Δ_1, i + 1_ - Δ_1, i_)	To detect rather regular but nonlinear patterns
	20. Mean |Δ_2_|	Useful for further classifying trajectories for that measure 19 is close to 0
	21. Max |Δ_2_|	Indicates whether there is an essential local peak or valley
	22. Ratio, max |Δ_2_|/mean-over-time	To assess the relative importance of the local peak
	23. Ratio, max |Δ_2_|/mean |Δ_1_|	To assess the relative importance of the local peak
	24. Ratio, mean |Δ_2_|/mean |Δ_1_|	To assess the relative importance of the fluctuation around the general rate of change
4. Measures contrasting early vs. later change	25. Ratio, early/later change	More remarkable than 1.0 if the magnitude of early change is more significant than the later change
	26. Ratio, early/total change	Share of early change from the total change
	27. Ratio, late/total change	Share of late change from the total change

We applied a factor analysis using the varimax rotation in the second step. The number of eigenvalues more than 1 specified the number of factors. Finally, a K-means algorithm clusters the factor scores. We specified the number of desirable clusters according to the Cubic Clustering Criterion (CCC) index and the flatten situation in the scree plot.

All data preparation, analysis, and data visualization were conducted by statistical software R version 4.0.5. In addition, we used the “traj” package in the R environment to cluster trajectories [32, 33]. This study was also conducted based on the Strengthening the Reporting of Observational Studies in Epidemiology (STROBE) Statement: guidelines for reporting observational studies. This checklist can be obtained from www.equator-network.org.

## Results

### Clustering incidence

**Fig. 1:**
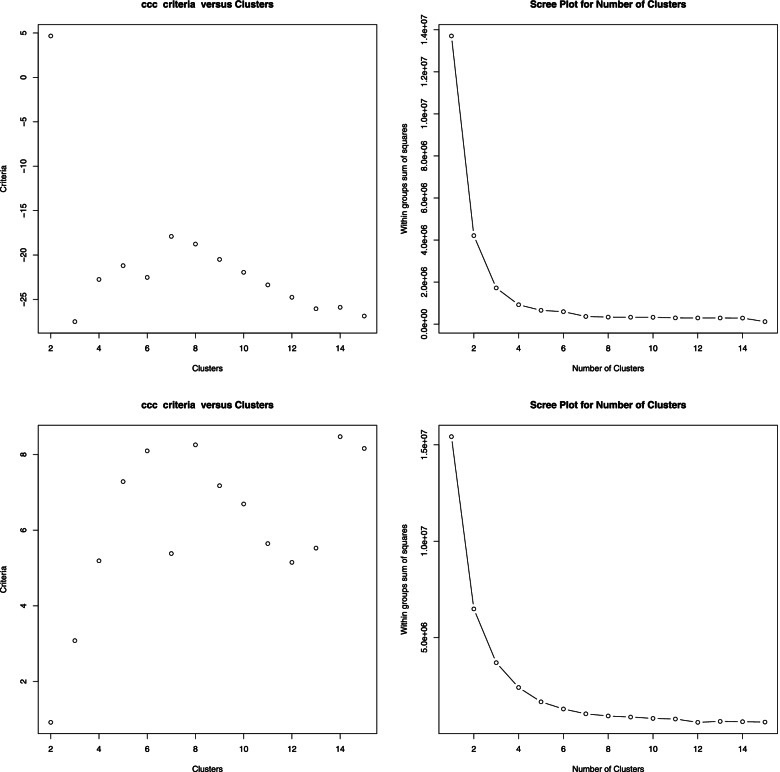
The Scree plot and the CCC criteria for detection number of clusters for weekly new cases and new deaths trajectories

According to Fig. 1, the scree plot starts to be flat in 3 clusters, and the within-group sum of the square does not decrease significantly. Therefore, we chose to put the trajectories in 3 clusters.

**Fig. 2:**
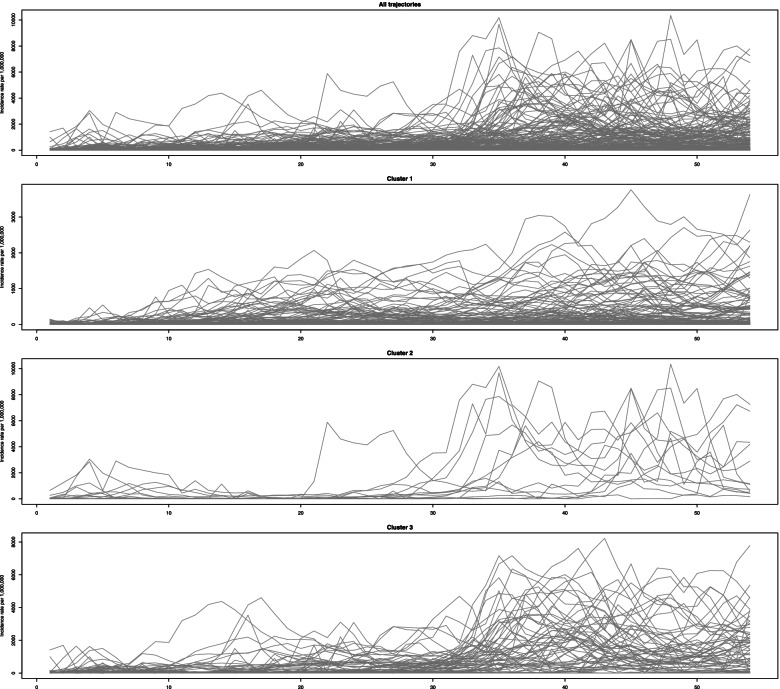
Observed trajectories of the incidence rate of COVID-19 per 1,000,000 population

Trajectories of COVID-19 incidence in the first year of the pandemic are presented in Fig. 2 for all clusters. Among 206 countries the 133 (64.56) countries belong to the second cluster, and 15 (7.28%) and 58 (28.16%) belong to the first and 3rd clusters, respectively. All clusters seem to be increasing in the study period, but there are several different patterns. In the first cluster, after 20 weeks, the incidence rate starts to be constant for almost 14 weeks, and after that increasing trend begins again. In the second cluster, we have a sharp increasing trend at the first 4 weeks, and then a decreasing trend starts and continues to week 20. After that, incidence increases sharply but in high variations. In the third cluster, the incidence rises smoothly until week 46 and, after that, decreases. Considering the magnitude of incidence, we could call the first cluster as Low, the second one as high, and 3rd one as the medium incidence clusters. We could call them mild, severe, and moderate increasing clusters in terms of the slope of increase.

**Fig. 3:**
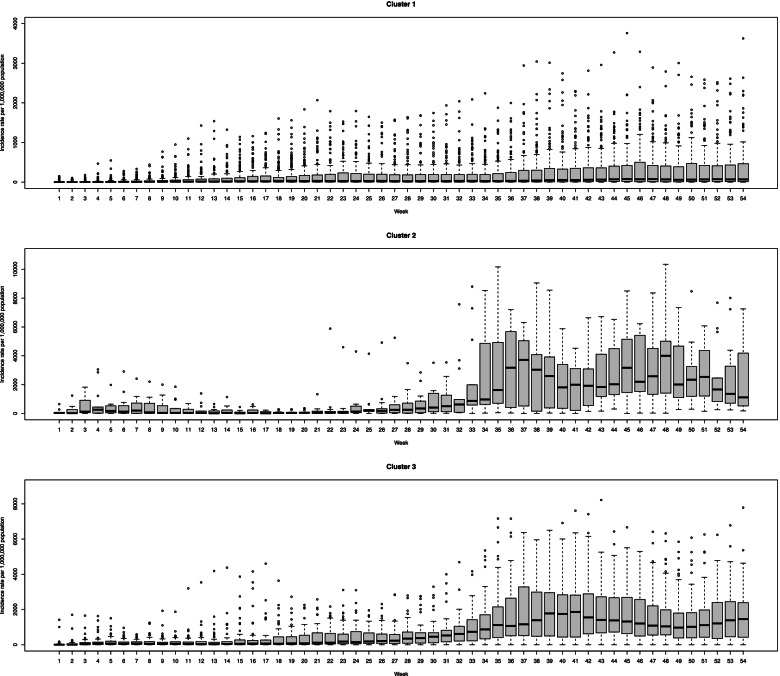
The box plots of incidence rate for three different detected clusters across weeks

Figure 3 represents the distribution of each cluster using the box plots. According to this figure, the most outlier is belonged to cluster one and weeks 11 to 18 of the 3rd cluster. Besides this, the points are homogenous within clusters. Generally, variation between countries after week 30 increased, and the incidence mainly increased in some countries.

### Clustering mortality

Interestingly, the scree plot and CCC index suggest the appropriateness of 3 clusters, the same as clustering the incidence rate (Fig. 1). In this manner, the frequency of sets is 37 (18.22%), 157 (77.34%) and, 9 (4.34%), respectively.

The data quality for incidence and mortality rate were different. As we mentioned earlier, the 206 countries and territories were used to cluster incidence rates. Among all these locations, the data of Vanuatu, Marshall Islands, Solomon Islands and, Samoa were not appropriate for clustering the mortality rate according to relatively missing values. On the other hand, the data of Gibraltar, which was not qualified for clustering incidence rate, was suitable for clustering mortality rate. Therefore, we used data from 203 countries and territories for this section.

**Fig. 4:**
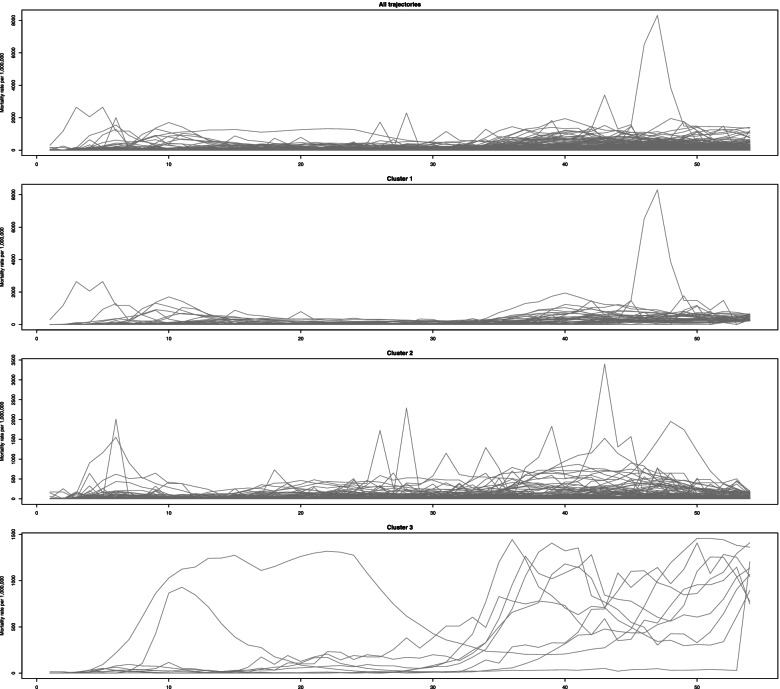
Observed trajectories of the mortality rate of COVID-19 per 1,000,000 population

The weekly new mortality rates trajectories for all the locations are depicted in Fig. 4. The highest peak in week 47 is related to Guinea by the mortality rate of 8309 per 1 million population. The mortality rates of weeks 46 and 48 are 6528 and 3857, respectively. Hence, there is a logical increasing trend, not excluding it.

**Fig. 5:**
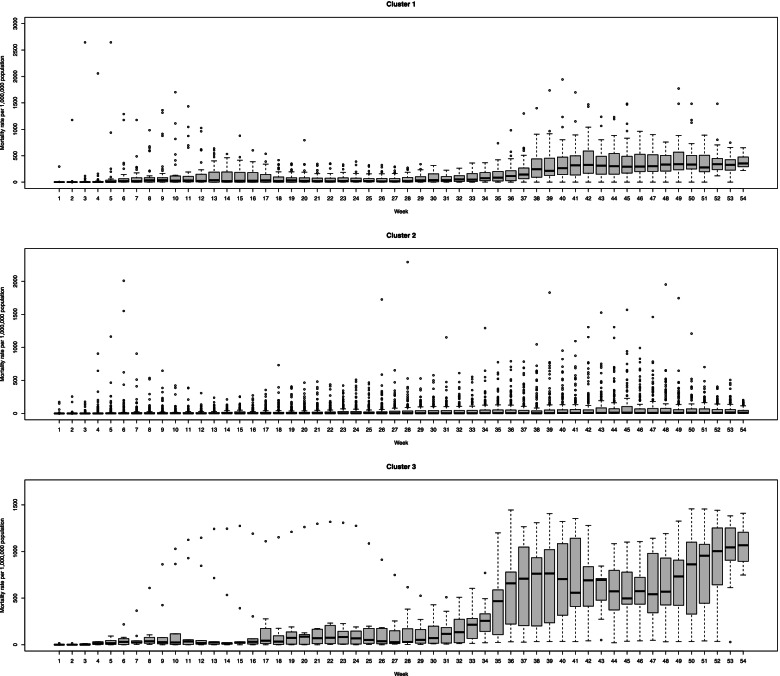
The box plots of mortality rate for three different detected clusters across weeks

All clusters have an increasing overall trend. The second cluster started to decrease after week 44, but 3rd cluster increased sharply after this time. The first two clusters experience several peaks before week 10, but the 3rd cluster only increases smoothly. Therefore, the first one increased moderately, the second one increased mild, and the 3rd one increased severely. As is shown in Fig. 5, the variation within clusters is lower at the initial of trajectories but increases over time.

### The geographical distribution of clusters

**Fig. 6:**
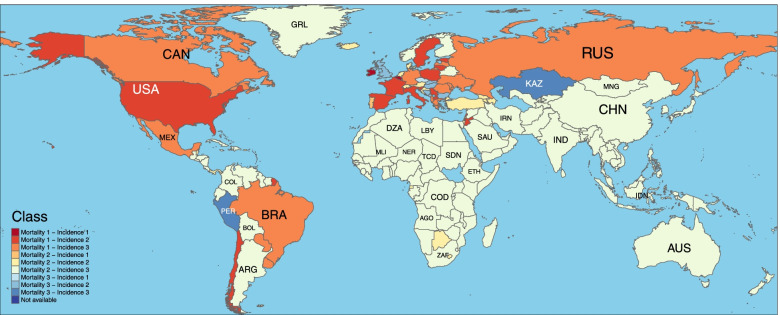
Cross classification of countries according to mortality and incidence clusters

The list of clusters based on nine possible combinations is available in Table 2 and the geographical distribution of these classes is presented in Fig. 6. Most countries that belong to the 3rd cluster of incidences are located in Europe and America. On the other hand, all members of the second cluster incidences are European countries. Therefore, the variation in Europe seems to be higher than in other areas of the world.

**Table 2:** Distribution of countries obtained by K-means algorithm on new cases and new deaths of COVID-19

**Mortality 1 (Moderate) - Incidence 1 (Mild)**	
Belgium, Isle of Man, Ireland, Mayotte, San Marino	
**Mortality 2 (Mild) - Incidence 1 (Mild)**	
Aruba, Andorra, Saint Lucia, Liechtenstein, Portugal, French Polynesia, Seychelles	
**Mortality 3 (Severe) - Incidence 1 (Mild)**	
Czechia	
**Mortality 1 (Moderate) - Incidence 2 (Severe)**	
Chile, Spain, Estonia, France, Italy, Jordan, Lebanon, Lithuania, Luxembourg, Monaco, North Macedonia, Malta, Poland, occupied Palestinian territory, including east Jerusalem, Serbia, Slovenia, Sweden, United States of America	
**Mortality 2 (Mild) - Incidence 2 (Severe)**	
Armenia, Austria, Azerbaijan, Bahrain, Belize, Bermuda, Barbados, Botswana, Switzerland, Cabo Verde, Cyprus, Denmark, Falkland Islands (Malvinas), Faroe Islands, Georgia, Guadeloupe, Equatorial Guinea, French Guiana, Guam, Croatia, Iceland, Israel, Lesotho, Maldives, Montserrat, Martinique, Netherlands, Panama, Puerto Rico, Qatar, Saint Pierre and Miquelon, Sao Tome and Principe, Turks and Caicos Islands, Turkey, Saint Vincent and the Grenadines, United States Virgin Islands	
**Mortality 3 (Severe) - Incidence 2 (Severe)**	
Bulgaria, Bosnia and Herzegovina, The United Kingdom, Hungary, Montenegro, Slovakia	
**Mortality 1 (Moderate) - Incidence 3 (Moderate)**	
Albania, Brazil, Canada, Germany, Greece, Latvia, Republic of Moldova, Mexico, Paraguay, Romania, Russian Federation, Ukraine, Uruguay	
**Mortality 2 (Mild) - Incidence 3 (Moderate)**	
Afghanistan, Angola, Anguilla, United Arab Emirates, Argentina, Antigua and Barbuda, Australia, Burundi, Benin, Burkina Faso, Bangladesh, Bahamas, Belarus, Bolivia (Plurinational State of), Brunei Darussalam, Bhutan, Central African Republic, China, Cameroon, Democratic Republic of the Congo, Congo, Colombia, Comoros, Costa Rica, Cuba, Cayman Islands, Djibouti, Dominica, Dominican Republic, Algeria, Ecuador, Egypt, Eritrea, Ethiopia, Finland, Fiji, Gabon, Ghana, Guinea, Gambia, Guinea-Bissau, Grenada, Greenland, Guatemala, Guyana, Honduras, Haiti, Indonesia, India, Iran (Islamic Republic of), Iraq, Jamaica, Japan, Kenya, Kyrgyzstan, Cambodia, Saint Kitts and Nevis, Republic of Korea, Kuwait, Lao People’s Democratic Republic, Liberia, Libya, Sri Lanka, Morocco, Madagascar, Mali, Myanmar, Mongolia, Northern Mariana Islands (Commonwealth of the), Mozambique, Mauritania, Mauritius, Malawi, Malaysia, Namibia, New Caledonia, Niger, Nigeria, Nicaragua, Norway, Nepal, New Zealand, Oman, Pakistan, Philippines, Papua New Guinea, Rwanda, Saudi Arabia, Sudan, Senegal, Singapore, Sierra Leone, El Salvador, Somalia, Suriname, Eswatini, Syrian Arab Republic, Chad, Togo, Thailand, Tajikistan, Timor-Leste, Trinidad and Tobago, Tunisia, United Republic of Tanzania, Uganda, Uzbekistan, Venezuela (Bolivarian Republic of), British Virgin Islands, Viet Nam, Yemen, South Africa, Zambia, Zimbabwe	
**Mortality 3 (Severe) - Incidence 3 (Moderate)**	
Kazakhstan, Peru	
**Mortality 1 (Moderate) - Incidence 1**	
Belgium, Isle of Man, Ireland, Mayotte, San Marino	
**Mortality 2 (Mild) - Incidence 1**	
Aruba, Andorra, Saint Lucia, Liechtenstein, Portugal, French Polynesia, Seychelles	
**Mortality 3 (Severe) - Incidence 1**	
Czechia

The geographical distribution of mortality clusters shows a similar pattern as we have already seen for incidence. Europe is divided into two clusters, and some countries belong to the same cluster as America. As a main result, most locations belong to the second cluster of mortality and the third cluster of incidences.

## Discussion

We determine that besides all variations in the country’s conditions, the spread pattern of a contagious disease follows from 3 different trajectories. Although these patterns are roughly similar, there are several specific features for each. In similar circumstances, we could not only focus on the general trend, but now we have an insight into little difference of trends. It is more efficient to investigate these features and pay attention to their value in the future variation of the disease’s trajectory. Interestingly the pattern of death from COVID-19 shows an agreement in the number of patterns with the incidence. This phenomenon is greater than the simple correlation between these two measures. Therefore, we suggest exploring the disease trend instead of simple measures like total cases and cumulative deaths. This approach could be helpful, especially for the communicable diseases that could lead to global disasters as a pandemic.

According to our findings, the typical pattern is the second cluster of mortality and the third cluster of incidences. Among 206 countries, 114 (55.33%) fit these clusters. The mortality trajectory of these countries exhibits several peaks in weeks 6, 26 (for some of them 28), and 43. Comparing this trajectory to others declares that death is highly related to the nature of epidemy. In addition, the variation within the trajectory is relatively higher than the other 2 clusters. The two possible reasons may be the registration quality and inefficient programs in these countries. Unfortunately, most of the countries in this class are characterized as low or middle-income ones.

On the other hand, the United States of America and central European countries like France, Spain and, Italy follow the first cluster of mortality and the second one of incidence. Variation of incidence trajectory probably shows the concerns in controlling the disease spread, but the consistency of mortality trajectory reveals the appropriate support of medical service to survive the patients.

Finally, the countries in the 3rd cluster of mortality and the first in incidence like Germany, Greece, Canada, Russian Federation, Ukraine and, Mexico seems successful in controlling the spread of disease more than achievement in surviving the patients.

In the Lai et al. study, the incidence and mortality of 57 countries from January 21, 2020, to February 29, 2020, were studied. The values of Spearman’s rank-order correlation between incidence and mortality rates per 1 million population and Daily Cumulative Index (DCI) were reported 0.667 and 0.452, respectively. This view is a simplistic aspect of the relationship between death and incidence. They ignored the trends and patterns. Therefore, their results do not apply to policymakers who should adjust their results consequently of new information. Another possible approach is to study trajectory patterns that we tried to conduct [34].

As a try in clustering patterns of COVID-19 trajectories, Zarikas et al. used hierarchical clustering of time series for 30 countries in the duration of starting epidemy and 80 days after that. They obtained four different clusters for the incidence of the disease. Their first and second clusters show the same trajectories pattern, increasing sharply and being constant. The only difference that we saw was in the level of incidence. According to their results, China belongs to the first cluster and South Korea to the second one. We refer both to our first cluster as their pattern becomes the same after the first 80 days (almost 12 weeks). In addition, the different level at the initial time was negligible compared to other countries, especially participants of the other two clusters [35].

There are several studies on the topic of clustering countries according to COVID-19. Melin et al. used self-organizing maps to cluster recovery cases, confirmed cases, and deaths related to COVID-19 and chose 4 clusters arbitrarily. Our results are relatively similar if we aggregate their low and medium clusters. For instance, Brazile, Russia, Mexico, Canada, and several middle European countries show the same pattern in both studies. In addition, the USA trend of mortality and incidence is higher than these countries [28].

Some studies used more future variables, which led to different clusters. Rizvi et al. explored data of 89 countries and 18 indicators of health systems and environmental conditions. Their K-means algorithm results in 4 clusters, but the role of economic-related variables in their work seems bold. Therefore, all high-income countries were assigned into a cluster regardless of the COVID-19 real trend [7]. In Zubair et al.’s study, a similar approach was made to detect countries with similar types of health care quality providers. In addition, they used the PCA method to handle the correlation between variables. This method notably decreased the within-cluster variation and improved the clustering algorithm. We conclude the same results in our study [27].

In contrast to mentioned studies, some studies applied highly correlated covariates like the proportion of countries population aged more than 65 years and the share of public health expenditure from the Gross domestic product (GDP). In this manner, Chandu found two significant clusters, so Australia, North American, and middle and west European countries as high case fatality rates versus all other countries (Chandu V: Identification of spatial variations in COVID-19 epidemiological data using K-means clustering algorithm: a global perspective, unpublished).

As another attempt to cluster the countries according to COVID-19, Pasin and Pasin compared the standard K-means method, obtaining the optimal clusters from the elbow and two-step clustering methods. The optimal cluster were 4 and 3 in these methods, respectively. The second approach significantly decreased the within clusters sum of the squares [36].

Another study in this domain was conducted for a 60 days duration by Mahmoudi et al. They chose seven very different countries and clustered them. Their limitation in selecting more countries was the complex fuzzy clustering method. Interestingly they found just three different patterns using a complex method. Following our results, central European countries have the same pattern as the United States, and East European countries are close to Asian, African, and South American countries [29].

The agreement of our results with the other studies, regardless of methodology or expanding study period, there are three significant patterns in an epidemy like COVID-19. The first and the worst one is not successful in managing the spread of the disease and the high level of mortality. The second one emphasizes more on the treatment of patients than the disease spread. The last one, focus on the disease in the first place and treat the patients. It seems the latest approach is more logical and successful.

Lots of data and information have been generated because of the importance of a pandemic in the new era. Unfortunately, some data has not an acceptable quality but using them still informs us. This study confronted some of these data types, shallow and middle-income countries. Despite all the efforts, there are still many issues in registration systems. Although this is a fundamental limitation, we ensure the reader that our method is highly robust against this issue.

We just aimed to extract the patterns and trajectories in our study. It seems another investigation on the details of the country’s policies is profoundly informative and essential. As this type of study could not manage a large sample, we recommend choosing the samples from our clusters as a starting point.

## Data Availability

This study used secondary aggregated data available for free access online by WHO. The information, user guidelines, and copyright rules are available on https://covid19.who.int/.
